# Evaluation of a conceptual framework for predicting navigation performance in virtual reality

**DOI:** 10.1371/journal.pone.0184682

**Published:** 2017-09-15

**Authors:** Jascha Grübel, Tyler Thrash, Christoph Hölscher, Victor R. Schinazi

**Affiliations:** Department of Humanities, Social and Political Sciences, ETH Zürich, Switzerland; University of Plymouth, UNITED KINGDOM

## Abstract

Previous research in spatial cognition has often relied on simple spatial tasks in static environments in order to draw inferences regarding navigation performance. These tasks are typically divided into categories (e.g., egocentric or allocentric) that reflect different two-systems theories. Unfortunately, this two-systems approach has been insufficient for reliably predicting navigation performance in virtual reality (VR). In the present experiment, participants were asked to learn and navigate towards goal locations in a virtual city and then perform eight simple spatial tasks in a separate environment. These eight tasks were organised along four orthogonal dimensions (static/dynamic, perceived/remembered, egocentric/allocentric, and distance/direction). We employed confirmatory and exploratory analyses in order to assess the relationship between navigation performance and performances on these simple tasks. We provide evidence that a dynamic task (i.e., intercepting a moving object) is capable of predicting navigation performance in a familiar virtual environment better than several categories of static tasks. These results have important implications for studies on navigation in VR that tend to over-emphasise the role of spatial memory. Given that our dynamic tasks required efficient interaction with the human interface device (HID), they were more closely aligned with the perceptuomotor processes associated with locomotion than wayfinding. In the future, researchers should consider training participants on HIDs using a dynamic task prior to conducting a navigation experiment. Performances on dynamic tasks should also be assessed in order to avoid confounding skill with an HID and spatial knowledge acquisition.

## Introduction

Researchers in spatial cognition have frequently relied on virtual reality (VR) in order to conduct experiments on human navigation [1, 2]. Some researchers have investigated the use of different human interface devices (HIDs; e.g., joystick, mouse and keyboard) with respect to navigation performance in virtual environments [3–6]. However, the specific aspects of spatial behaviour that mediate the relationship between skill at manipulating the HID and navigation performance have yet to be determined. Interaction with an HID may be related to navigation differently than natural walking through a real environment because the HID involves an additional layer of abstraction between an intended action and its perceptual consequences [7, 8]. This mapping between action and perception may be learned incrementally in a similar way as movements in real environments [9], but people generally have more experience with natural walking than with manipulating an HID. In addition, experience with a specific HID may explain performance differences for various navigation tasks [3, 6]. The present study assesses the manner in which participants’ skills with an HID relates to navigation performance in a virtual environment.

According to Montello [10, p. 258-260], navigation can be decomposed into locomotion (i.e., manoeuvring through a large-scale environment) and wayfinding (i.e., spatial decision-making). Traditionally, spatial cognition research has focused on the importance of spatial memory for wayfinding tasks and may have overlooked the importance of locomotion for large-scale navigation. Following Gibson [11], Heft [12] has characterized the process of navigation as apprehending the invariant structure of an environment during locomotion through a sequence of vistas (i.e., the features available to an observer from a particular viewpoint) separated by transitions (i.e., points along a route at which a previously occluded vista gradually comes into view). However, there is insufficient evidence to suggest that a locomotion-based theory can explain navigation more generally (but see [13]).

Spatial behaviour has also been characterised along other dichotomies, including perception-action and cognitive components [14–17], fine-grained and categorical spatial representations [18–20], coordinate and categorical spatial representations [21–24], taxon and locale systems [25–28], online and offline processes [29], and egocentric and allocentric reference frames [30]. Allen and Haun [31] have ascribed some of these distinctions to the same two spatial processing systems but note that alternative theories with more systems may be appropriate (cf., [8]). Rather than presuming the alignment of different two-systems theories, the framework used for the present study constructs several orthogonal dimensions based on existing systems in order to predict navigation performance. These dimensions consist of static and dynamic stimuli, perceived and remembered information, egocentric and allocentric reference frames, and distance and direction judgements.

In VR, the user tends to be dynamic, but distinct stimuli (i.e., buildings, trees) in the virtual environment can be either static or dynamic. For example, a parked car can be considered a static stimulus, and a car moving down the street can be considered a dynamic stimulus. With respect to optic flow, static stimuli result in invariant spatial information in the visual field relative to their surroundings [11]. In contrast, dynamic stimuli can move through the visual field independently of changes in optic flow that result from self-motion [11]. Previous research in VR has often employed static stimuli in order to investigate navigation [1]. These studies have successfully demonstrated the role of spatial memory for navigation through static environments. For example, spatial memory may be assessed in terms of participants’ abilities to shortcut [32], build models [33], and conduct judgements of relative direction [34] However, the focus on static environments may have resulted in a bias towards tasks that rely on the integration of spatial information over time in memory [35, 36] and neglected the potential importance of dynamic stimuli perceived during navigation ([37]; but see [38, 39]). Responses to dynamic stimuli in VR may require more skill at manoeuvring the HID than responses to static stimuli when the stimuli move in an unpredictable manner. Thus, tasks with dynamic stimuli may tap previously unidentified individual differences in locomotion behaviour during navigation.

The static/dynamic dimension may also be disentangled from a perceived/remembered dimension during navigation because the perception (and not necessarily representation) of static objects is critical for many spatial behaviours [33, 40, 41]. Indeed, these spatial behaviours are often used to infer differences in mental representations but could also indicate difference in the initial perception of the objects, even when they are no longer visible. This distinction between perceived and remembered spatial information has important consequences for spatial reasoning with respect to immediate and remote environments [14, 42], especially when perception and memory are considered along a continuum. In this context, recently learned environments would lie between immediate and remote environments. For example, Waller and Hodgson [42] found that the representation of a remote environment can be relatively less accurate and less precise than the representation of an immediate environment [42]. However, in aggregate, less precise representations may lead to more accurate localisations [18]. On the other hand, responses to recently learned information tend to be relatively more precise [42] and more accurate (depending on response modality; [14]) than very familiar information.

According to Avraamides and Kelly [29], perceived information typically involves an egocentric reference frame, but remembered information may be egocentric (e.g., during scene recognition; [43]; [44]; as during pointing responses; [45]) or allocentric (whether intrinsic; [46]; or environmental; [47]; for a review, see [30, 48]). However, some researchers claim that remembered information is also primarily egocentric [49, 50]. During navigation, individuals may rely on external representations that are either egocentric (e.g., route instructions; [51]) or allocentric (e.g., maps; [52]) and may be employed to enforce a navigator’s choice of reference frame. The ease with which one uses either type of external representation during navigation can also indicate the format of the corresponding internal representation [52].

Distance and direction estimates have been used to infer egocentric representations based on a static, perceived environment (e.g., [14, 40, 53]); egocentric representations based on a static, remembered environment (e.g., [14, 42, 45, 47, 53–55]); egocentric representations based on a dynamic, perceived environment (e.g., [36, 38, 39]); and allocentric representations based on a static, remembered environment (e.g., [42, 47, 52, 54, 55]. In addition, distance and direction judgements may also reflect two different spatial abilities because of differences in how translations and rotations are perceived and remembered (e.g., [4, 40, 56–59]). For example, Easton and Sholl [56] found that rotations and translations led to different performance profiles in regularly (but not irregularly) structured arrays of objects. Thus, this distinction between direction and distance may represent an additional dimension of spatial task and may be orthogonal to static/dynamic, perceived/remembered, and egocentric/allocentric dimensions.

The present study investigates the manner in which these four orthogonal dimensions of spatial tasks can be used to predict navigation through a virtual environment. Specifically, we expect tasks with dynamic stimuli to be the best predictors of navigation behaviour in a familiar virtual environment because these tasks are closely associated with participants’ skills when using an HID. Towards this end, we designed eight simple tasks that systematically assess different points along these dimensions. We related performance on these eight tasks to navigation through a virtual reality replica of a university campus [33]. To anticipate, we found that an egocentric task in which participants chased a moving object predicted goal-directed navigation better than all four dimensions taken together.

## Methods

### Participants

Twenty-three participants were recruited for the experiment from the University Registration Center for Study Participants (https://www.uast.uzh.ch/) via the ETH Decision Science Laboratory (DeSciL). Three participants (two female) experienced simulator sickness and were excluded from the analyses. Of the remaining 20 participants, 11 were female. The age of the participants ranged from 18 to 28 years (*M* = 21.8, *SD* = 3.01).

#### Ethics statement

The experiment was approved by the ETH Zurich Ethics Commission (EK 2013-N-73). Prior to starting the experiment, written informed consent was obtained from all participants. The participants were paid 30 CHF per hour. Participants that aborted the experiment due to simulator sickness were compensated with 20 CHF.

### Materials

#### Hardware

The technical setup for the experiment consisted of a WorldViz CAVE setup with three computers. Each system was equipped with a Core i7-3820 at 3.6 GHz with 12 GB of RAM and an Nvidia Quadro K4000 with 3 GB RAM. The CAVE consisted of three ultra short throw projectors NEC U310W running at a 1680 x 1050 resolution during 3D projection. To enable 3D perception, alternate frame sequencing shutter glasses of the type *Volfoni 3DGE RF* were used. The *WorldViz PPT Real-Time Motion Tracking System* was used for tracking head position and orientation. The tracking system was connected to a separate computer to reduce the computational load on the main machines. Participants were seated in a chair that was located in the middle of the CAVE facing towards the middle screen. A small table was mounted on the arm rests to comfortably place the joystick (Logitech Extreme 3D Pro) for the participants.

The motion sensors attached to the participants’ head provided the orientation of the participant in relation to the CAVE and was used to determine the participants’ orientation in the virtual environment. The head orientation together with the joystick was used to turn and move within the virtual environment. Translational movements were executed by pushing the joystick in the desired direction (i.e., forward, backward, left, and right) while rotations were performed by twisting the joystick left or right and turning the head. However, there was a subtle difference in how the joystick and head trackers were used to control rotation. When using the joystick, the projected virtual environment rotated to display the desired view direction. In contrast, turning the head merely changed the virtual direction from which we recorded the observer’s viewing direction. A visual “catchment area” was provided in order to facilitate the interaction with elements in the environment. This catchment area consisted of a yellow semi-transparent circle on the ground that moved with the participant’s position and head rotation (yaw axis) to indicate the location where we consider an interaction to occur. All translational movements were performed relative to the viewing direction (i.e., pushing the joystick forward always resulted in the expansion of optic flow from the point of focus).

#### Software

We used custom-designed software [60] for conducting experiments with a Vizard CAVE system. This software provided automatic data storage (i.e., logging the position of the observer and static/dynamic elements) and logic units to setup the experiment. The obtained data was stored in a MySQL database (version 5.6.16) and subsequently exported to Matlab 8.2.0.29 (R2017a) for further processing and analysis.

#### Virtual environments

**Fig 1:**
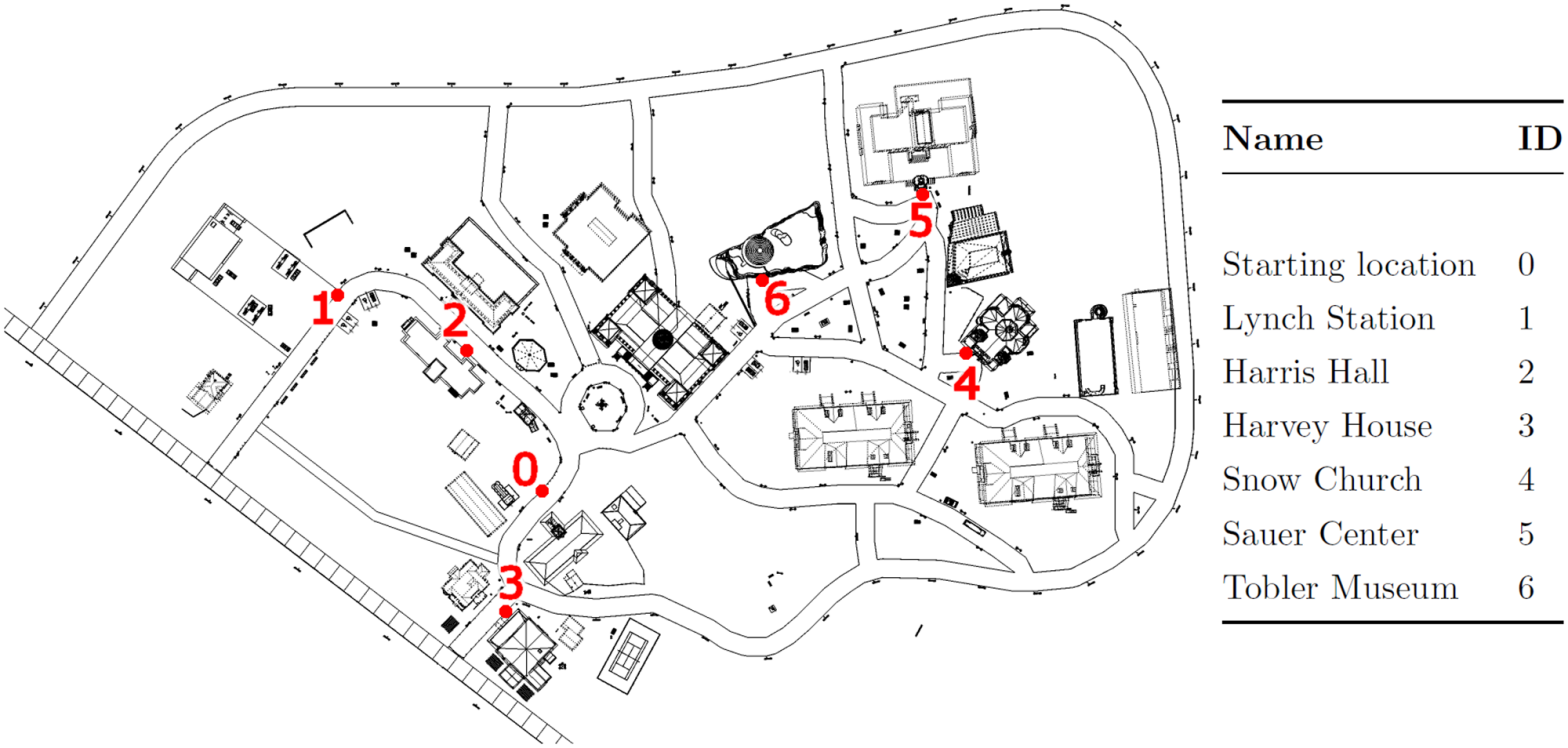
Overview of the Virtual SILCton Environment and the target locations. A top-down perspective of Virtual SILCton with the six target locations (red). The ID of each location does not correspond to the order of visits during the experiment.

Two different virtual environments were used in this experiment. One environment (the Sphere Environment) consisted of a small meadow (40 meters x 40 meters) with randomly placed spheres. Each sphere had a radius of 0.25 meters, floated 0.25 meters above ground, and had a minimum distance of 2 meters to the nearest sphere. The other environment (the Virtual SILCton Environment) consisted of a small road network, 22 buildings, and some additional structures (e.g., statue, benches). Six locations were selected for the navigation task. A sign with each location’s name was placed in front of each target (Fig 1). The digital model of Virtual SILCton has been used in previous spatial navigation research [33]. The model was originally created in Sketchup and then exported to Vizard as a collada file.

### Procedure

Upon arriving at the lab, participants were presented with a document describing the main goals and experimental procedure (see S1 File on page 21) and were asked to complete the consent form. Participants then completed the Santa Barbara Sense of Direction scale (SBSOD) [61]. Before each phase, they were given written instructions regarding each of the VR tasks (see S1 File). A small set of written questions was also given to the participants in order to ensure that they read and understood the instructions. At this stage, participants were also given time to ask questions about the experiment and procedure. Participants were then seated in the middle of the CAVE and given the joystick. The full protocol of the experiment is available online on protocol.io [62].

Participants completed a Training phase in the Sphere Environment, a Navigation Phase in the Virtual SILCton Environment and a Simple Tasks Phase in the Sphere Environment. During the training task, participants were also allowed to ask questions regarding the joystick and follow-up tasks but were asked to refrain from asking questions during testing. A video representing the entire experimental procedure is available online as supplementary material (see S1 Video).

#### Training Phase

**Fig 2:**
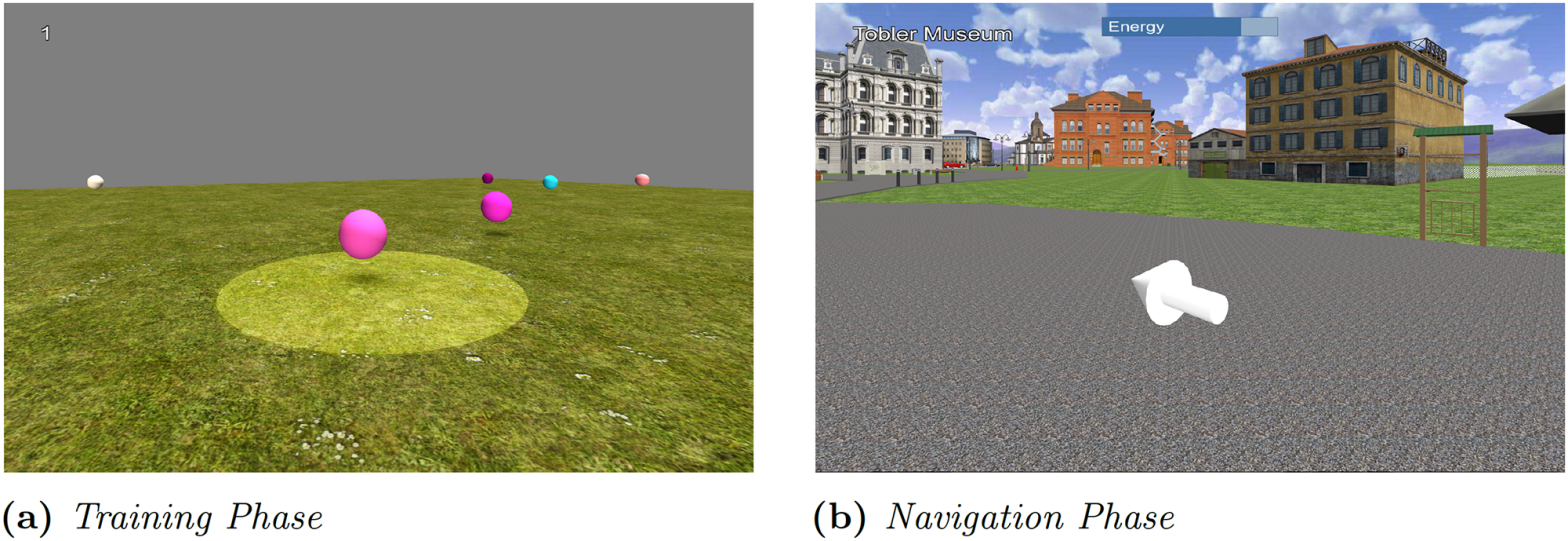
Training and navigation. (a) Screenshot of a participant collecting a sphere during the training phase. The yellow catchment area surrounds the intended target. For this figure, the catchment area appears slightly brighter than in the actual experiment. (b) Screenshot of a participant calling the arrow during the navigation phase. The destination (Tobler Museum) is indicated in the top-left corner of the screen. The energy bar is placed at the top of the screen.

The training phase was used to familiarise the participants with the VR setup and joystick. Participates were asked to use the joystick to move around and collect 10 of 40 randomly coloured and placed spheres. The visual “catchment area” was provided in order to facilitate the collection task (Fig 2a). To collect a sphere, participants were asked to place the sphere within the catchment area and press the trigger button on the joystick. A counter at the top of the screen indicated when they collected a sphere.

#### Navigation Phase

In this phase, participants were asked to find a series of goal locations in the Virtual SILCton environment. Participants were unfamiliar with this virtual environment at the beginning of the Navigation Phase, so the first block of trials constituted a search task. During navigation, participants could press the trigger button on the joystick in order to call up a 3D arrow that pointed in the straight-line direction of the target locations (ignoring any potential obstacle along the way). The arrow did not guide the participants along a predefined route to the target location. An energy bar was used to limit participants’ interactions with the arrow (Fig 2b). Energy was consumed as the participants pressed the trigger. When the energy was depleted, participants were required to wait 10 seconds before they could trigger the arrow again. This mechanism prevented participants from continuously pressing the trigger but allowed them to use it primarily when they were disoriented.

The process of visiting all target locations was repeated over four blocks. During each of the first three learning blocks, participants were asked to visit the six locations in a random order. At the beginning of each trial, a large text appeared at the centre of the middle screen of the CAVE that indicated the name of the destination. Once displayed, the name of the destination remained at the top-left corner of the middle screen until participants reached the destination. During the fourth testing block, participants were asked to find the six target locations but without the help of the arrow. During testing, the visiting order of the target locations was fixed. This fixed order was designed to allow for comparisons across participants.

#### Simple Tasks Phase

Participants performed a set of eight different tasks in random order in each of five blocks. Twenty white floating spheres were used for each of the simple tasks. For each task, target spheres were coloured blue. A pause screen appeared before each task and displayed a short description of the upcoming task. After each task, participants were rotated by a random angle and the spheres moved to new random locations. Participants used the joystick trigger to indicate that they completed the task to the best of their ability. No other feedback was provided to the participants. The name of the current task was displayed at the top-left corner of the main screen. Similar to the training task, a catchment area indicated the participants’ positions and head directions. For some tasks, an additional top-down map of the environment was displayed at the top-right corner of the middle screen that occupied 20% of the width and height of that screen.

**Fig 3:**
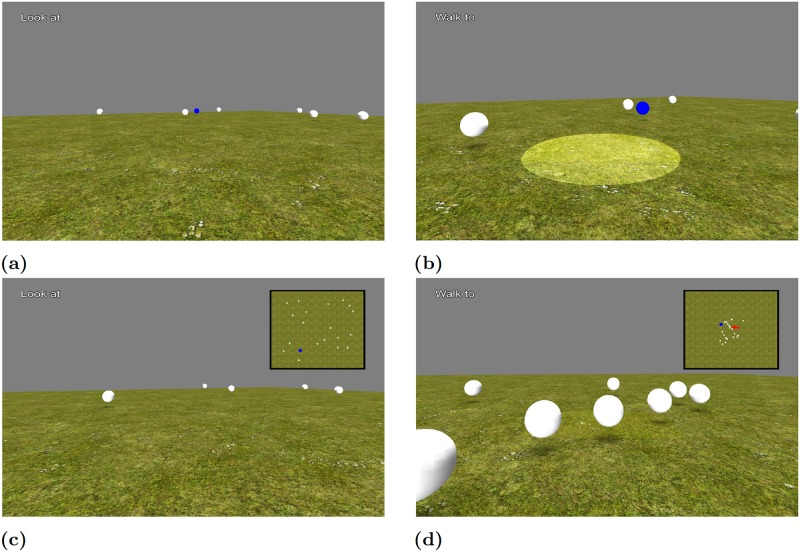
Simple task example trials. Images representing examples of the different tasks from the participant’s perspective. (a) Image of the Rotate (ROT) task from a first-person perspective. (b) Image of the Move (MOV) task from a first-person perspective. (c) Image of the Rotate with map (RWM) task from a first-person perspective with a north-up, top-down map. (d) Image of the Chase with map (CWM) task from a first-person perspective with a north-up, top-down map. This selection exhibits different components present in all tasks. For this figure, the catchment area appears slightly brighter than in the actual experiment and the size of the spheres on the map has been increased to be more visible to the reader.

Below are descriptions of each of the eight simple tasks. Fig 3 includes images of selected exemplary tasks.

**Rotate (ROT):** Participants were asked to rotate to a target blue sphere. A successful trial consisted of turning until the blue sphere was in front of the participant’s head. Translations were disabled throughout this task.

**Move (MOV):** Participants initially faced a target blue sphere and were asked to walk towards it as accurately as possible.

**Rotate with map (RWM):** A north-facing, top-down map was displayed at the top-right corner of the middle screen. This map did not provide any indication of the participant’s position in the virtual world. Participants were asked to turn towards the target sphere that was coloured blue on the map. The white spheres were also visible on the map. The target sphere was blue only on the map and was not visibly distinguishable from the other (white) spheres from the first-person perspective. Translations were disabled throughout this task.

**Move with map (MWM):** A north-facing, top-down map was displayed at the top-right corner of the middle screen. The map did not provide any indication of the participant’s position in the virtual world. Participants were asked to walk to the location of the blue-coloured target sphere on the map. The white spheres were also visible on the map. The target sphere was blue only on the map and was not visibly distinguishable from the other (white) spheres from the first-person perspective.

**Rotate from memory (RFM):** Participants were asked to rotate sequentially to two blue target spheres. After the second rotation, all the spheres disappeared, and participants were asked to rotate back towards the direction of the first target sphere. Translations were disabled throughout this task.

**Move from memory (MFM):** Participants started the task facing a blue-coloured target sphere. Once participants started moving, all the spheres disappeared. Participants were asked to stop moving when they reached the previous location of the target sphere.

**Chase (CHA):** All spheres moved randomly within the virtual field. Participants were asked to move and intercept the blue target sphere as quickly as possible.

**Chase with map (CWM):** All spheres moved randomly within the virtual field. A north-facing, top-down map was displayed at the top-right corner of the middle screen. The map indicated the participant’s position in the virtual world with a red arrow. The location on the map was continuously updated. Participants were asked to move and intercept the blue target sphere shown on the map. The white spheres were also visible on the map. The target sphere was blue only on the map and was not visibly distinguishable from the other (white) spheres from the first-person perspective.

### Design and analysis

The eight simple tasks were designed to represent different combinations of the static/dynamic, perceived/remembered, egocentric/allocentric, and distance/direction dimensions described above.

**Static versus Dynamic Stimuli**. In each task with static objects, no spheres in the environment could be moved or move on their own. In contrast, tasks with dynamic objects contained spheres that moved independently of participants’ actions.

**Perceived versus Remembered Information**. Each task was defined as to whether participants could complete the task based on the immediate environment or based on a mental representation of the environment.

**Egocentric versus Allocentric Reference Frame**. Tasks that emphasised egocentric reference frames only presented information from a first-person perspective. In contrast, tasks that emphasised allocentric reference frames included a map of the environment from a top-down perspective.

**Direction versus Distance**. Tasks were also defined as to whether participants performed translations or rotations towards the target sphere.

#### Task selection

**Fig 4:**
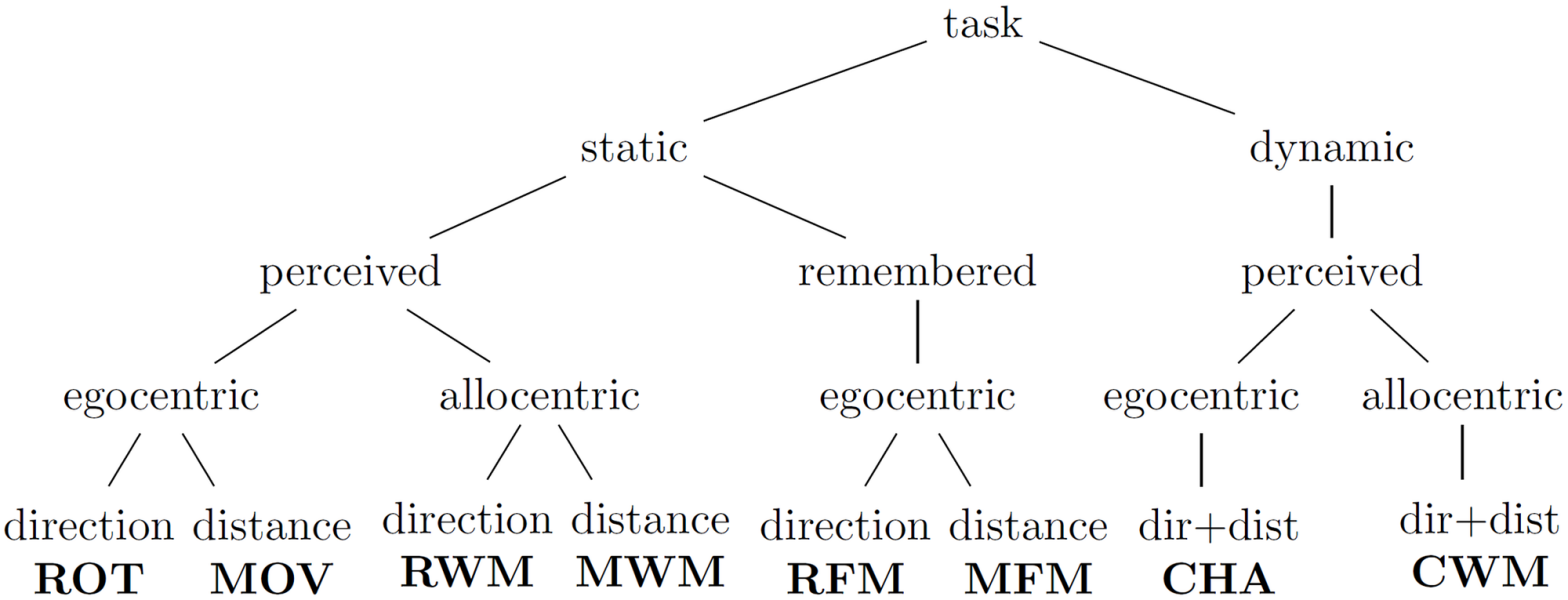
Task classification tree. This tree represents the variable assignments for each of the eight tasks. Independent variables are inner nodes, and tasks are presented as leaves.

The relationships among the eight simple tasks in terms of the four orthogonal dimensions can be visualised as a tree (see Fig 4).

Out of 16 possible variants of the four orthogonal dimensions, eight variants are not suitable. First, the combination of *dynamic* stimuli and *remembered* information is not suitable because it is unclear how participants could predict the movement of a randomly moving sphere. Second, the combination of *remembered* information and *allocentric* reference frame is not suitable because participants could use either egocentric or allocentric mental representations to complete the task.

#### Measurements

Participants’ performances in the Navigation and Simple Task Phases were measured with respect to the time required to complete each task and deviation in terms of angle and distance from the correct path. For the Navigation Phase, this required logging of the participant’s position and orientation within the virtual environment and the ID of each location in the scene. We also recorded the number of trigger presses (calling the arrow) as a measure of learning during navigation. For the Simple Task Phase, we logged the participants’ positions, orientations, and trigger presses (indicating task completion). Here, we also logged the position(s) of the sphere(s) with which participants were interacting. Over 600,000 data points were collected throughout the experiment and were directly logged into the database.

#### Analysis

Data from the SBSOD and virtual environments was imported to Matlab and SPSS for analysis. For details on the database, refer to S1 Data. In a first preprocessing step, the raw data was grouped by participant and experiment scene. This data was then split according to indicator variables that marked the beginning and end of each task. For the dynamic sphere tasks, the data points were resampled at a fixed time step to obtain uniform samples. Weighted linear interpolation between two objectively measured points was used to obtain a complete sample at the required time steps (see S1 Code). We next computed error measures for both Navigation and Simple Task Phases. We also conducted a Regularised Exploratory Factor Analysis (REFA) [63, 64] for assessing the relationships among the various tasks and attempted to predict navigation performance using both the four orthogonal dimensions and the REFA factors. Additional statistical analyses were performed with SPSS (see S2 Data)

#### Task errors

As a metric for performance in the Navigation Phase, we used ArcGIS [65] to measure the optimal route distance *d*_*r*_ between target locations and compared them to the actual distances *d*_*p*_ walked by participants in the virtual environment. The ratio *r*_*d*_ was considered the error measure as shown in Eq (1).

$$\begin{array}{c} r_{d}=\frac{d_{p}}{d_{r}} \end{array}$$ (1)

Four error measures were devised to account for the participants’ overall performance and their accumulated error within each of the eight simple tasks. Good performance was indicated by a score close to or equal to 0, and bad performance was indicated by a score close to or equal to 1.

Error measures were deviations in either rotation or distance from the optimal choice. To score the performance at the end of a static task, we computed the *final* deviation to the optimal outcome (e.g., looking in the target direction or standing at the target location). Scores on the dynamic tasks were computed by *accumulating* error at each time step based on whether the participants’ action was optimal (e.g., bringing them closer to view the target direction or moving them closer to the goal location; see S1 Code).

In order to calculate the final error of a participants’ rotation, we defined the absolute value for the angle *ε*_*r*_ between the participants’ viewing direction *α*_*p*_ and the direction towards the goal from their location *α*_*g*_. In addition, we mapped degrees onto the interval [0, 1] as shown in Eq (2). The error measure semantically defines 0 as looking directly at the goal and 1 as looking in the exact opposite direction of the goal.

$$\begin{array}{c} \varepsilon_{r}=\frac{abs(\alpha_{p}-\alpha_{g})}{180} \end{array}$$ (2)

To measure a term *ε*_Δ*rt*_ of the cumulative direction error *ε*_Δ*r*_, a simple *sign function* *δ*_*r*_ returned 1 if the participants rotated towards the goal, −1 if they turned away from the goal, and 0 if they remained static. Here again, we map the *sign function* results onto the interval [0, 1]. This refers to the rotation in degrees that participants performed between two sequential measures in time [*t*, *t* + 1] as shown in Eq (3). To obtain the cumulative direction error, the direction error at each time step *ε*_Δ*rt*_ is summed and divided by the number of time steps *T* in a task, as shown in Eq (4).

$$\begin{array}{c} \varepsilon_{\Delta rt}=\frac{\delta_{tr}+1}{2}\Delta \alpha_{p} \end{array}$$ (3)

$$\begin{array}{c} \varepsilon_{\Delta r}=\frac{1}{T}\sum_{t=1}^{T}\varepsilon_{\Delta rt} \end{array}$$ (4)

The final distance error is the ratio of the participant’s start distance to the goal *d*_*s*_ (i.e., the location at the beginning of the task) to their end distance to the goal *d*_*e*_ (i.e., the location at the time when the participant pressed the trigger to indicate the completion of a task). The starting point refers to the participants’ location at the beginning of the task, and the end point refers to the participants’ location at the time when they pressed the trigger indicating that they completed the task. In addition, an offset of *δ*_*c*_ = 4*m* (equivalent to the distance between the participant and the centre of the catchment area) was used to account for the catchment area. The resulting error measure *ε*_*d*_, as shown in Eq (5), was also mapped onto the interval [0, 1]. An error of 1 indicated that the participants kept a distance equal to or larger than the start distance *d*_*s*_ to the goal. An error of 0 indicated that a participant reached the goal up to the precision of the catchment area.

$$\begin{array}{c} \varepsilon_{d}=min(1,abs(\frac{d_{e}-\delta_{c}}{d_{s}-\delta_{c}})) \end{array}$$ (5)

To measure the term *ε*_Δ*dt*_ in the cumulative distance error *ε*_Δ*d*_, the optimal distance *d*_*opt*_ that participants’ could have reached with Δ*d*_*p*_ was compared to the actual distance $d_{p}^{\prime }$ that they reached in the following time step. Here, Δ*d*_*p*_ refers to the distance in meters that the participant moved between two sequential measures in time. The results are mapped onto the interval [0, 1] by dividing by 2Δ*d*_*p*_ (see Eq (6)). To obtain the cumulative direction error, each *ε*_Δ*dt*_ is summed up and divided by the number of time steps *T* in a task (see Eq (7)).

$$\begin{array}{c} \varepsilon_{\Delta dt}=\frac{d_{p}^{\prime }-d_{opt}}{2\Delta d_{p}} \end{array}$$ (6)

$$\begin{array}{c} \varepsilon_{\Delta d}=\frac{1}{T}\sum_{t=1}^{T}\varepsilon_{\Delta dt} \end{array}$$ (7)

#### Regularised Exploratory Factor Analysis

Developed by Jung and Takane [63], Regularised Exploratory Factor Analysis (REFA) can be used with small sample sizes (*n* < 50) that may cause erratic behaviour in other types of Exploratory Factor Analysis (EFA) or Principal Component Analysis (PCA). With small sample sizes, the sample covariance matrix tends to be near singular and numerically ill-conditioned, which makes the application of EFA difficult. Furthermore, PCA is not always appropriate because it does not model measurement errors [64, 66]. For REFA, it is assumed that the unique variance *Ψ* is proportional to a tentative estimate of *Ψ*. This estimate is adjusted via the regularisation parameter *λ* [63]. For the present study, we adopted the one-parameter maximum likelihood (ML) estimation method under the anti-image assumption (ML REFA) [64]. ML REFA produces better results for small samples than other approaches [63] including unbiased estimates of factor loadings, smaller standard deviations, and smaller mean squared errors (MSEs). To estimate the number of factors, permutation tests (equivalent to parallel analysis) were employed [64, 67]. The resulting factors were then rotated using an oblique geomin rotation [68].

We applied REFA in order to identify the underlying factors of participants’ performance in the eight tasks. For each of the eight simple tasks, a standard score *zp*_*i*_ was aggregated for the five repetitions. The error *ε*_*p*_*i*__ (see Eq (8)) was used to compute the standardised score (see Eq (9)). For purely directional tasks, the sum of the final direction error and cumulative direction error equals zero. Thus, those tasks were divided by 2 rather than 4.

$$\begin{array}{c} \varepsilon_{p_{i}}=\frac{\varepsilon_{r}+\varepsilon_{\Delta r}+\varepsilon_{d}+\varepsilon_{\Delta d}}{4} \end{array}$$ (8)

$$\begin{array}{c} z_{p_{i}}=\frac{\varepsilon_{p_{i}}-\mu }{\sigma } \end{array}$$ (9)

We used the standardised scores across all eight tasks as input to the REFA Matlab library provided by [64] and computed communalities to assess the quality of the factor analysis. The communality *h*_*i*_ indicates the variance of a task *i* explained by the loading *l*_*j*_ *i* in all *m* factors [69] (see Eq (10)). We then computed the total communality *h*_*t*_ (see Eq (11)) and the mean communality *h*_*m*_ (see Eq (12)) that indicate the total variance that the factors can explain.

$$\begin{array}{c} h_{i}=\sum_{j=1}^{m}l_{ji}^{2} \end{array}$$ (10)

$$\begin{array}{c} h_{t}=\sum_{j=1}^{m}h_{i} \end{array}$$ (11)

$$\begin{array}{c} h_{m}=\frac{h_{t}}{m} \end{array}$$ (12)

## Results

The results are divided into three sections. First, we present the results of the Navigation and Simple Tasks Phases. Then, we relate performance from the Simple Tasks Phase to performance in the Navigation Phase using both REFA and regression analysis.

### Navigation Phase

Given that we deliberately randomised the order of trials during learning (but not testing), we could not compare navigation performance across blocks in terms of time or deviations from the optimal path. To test for learning in the Navigation Phase, we performed a repeated measures ANOVA with a Greenhouse-Geisser correction [70] for a violation of sphericity and found a difference among the three blocks in terms of the number of trigger presses, (*F*(1.39, 26.35) = 53.86, *MSE* = 2324.42, *p* < .001). Two-tailed pairwise contrasts revealed significant differences between trigger presses in block 1 (*M* = 24.15, *SD* = 8.88) and block 2 (*M* = 12.10, *SD* = 7.75; *F*_1,19_ = 40.48, *MSE* = 71.73, *p* < .001, *d* = 1.45) and between trigger presses in block 2 and block 3 (*M* = 6.60, *SD* = 7.38; *F*(1, 19) = 28.81, *MSE* = 21.00, *p* < .001, *d* = 0.73).

Participants required a mean of 65.69 seconds (*SD* = 4.85) to complete the testing block with a mean distance error ratio of 1.13 (*SD* = 0.09). A two-tailed, one-sample t-test comparing the average distance error ratio to one revealed a significant difference (*t*_19_ = 6.86, *se* = 0.02, *p* < .001, *d* = 1.53).

The two-tailed correlation between SBSOD and mean distance error ratio from the testing block was not significant (*r*_18_ = .22, *p* = .35).

**Fig 5:**
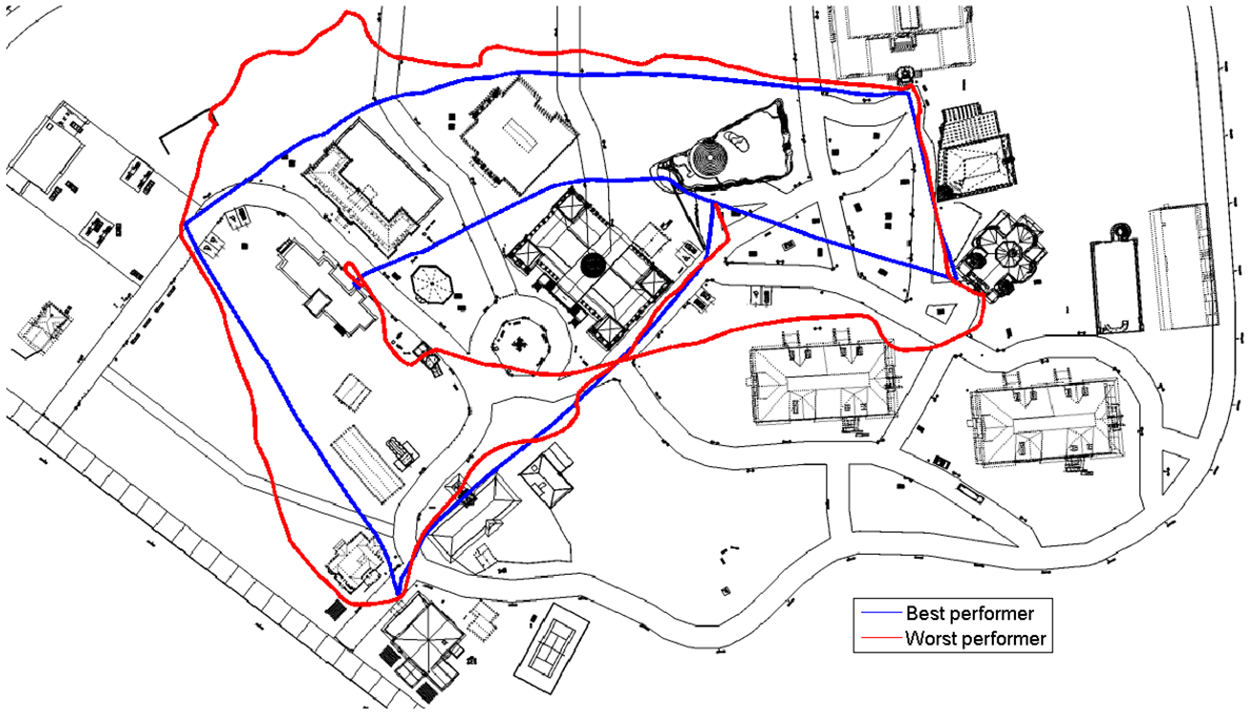
Paths of best and worst participants during testing. The blue line traces the path of the best-performing participant. No wrong turn was taken and nearly no deviations occurred on straight paths. The red line traces the path of the worst-performing participant. Many wrong turns and unnecessary deviations from straight paths can be observed.

Fig 5 presents the best and worst performing participants’ routes in the testing phase.

### Simple Tasks Phase

In order to obtain a better estimate of participants’ performances for the eight simple tasks, we consider performance aggregated across all trial types (see Table 1).

**Table 1:** Descriptive statistics for simple tasks.

Task	Performance			Time		
	Mean	Median	SD	Mean	Median	SD
Rotate	.203	.186	.069	7.00	6.81	1.73
Move	.074	.024	.078	8.73	8.57	2.05
Rotate with map	.186	.180	.101	50.08	49.03	1.73
Move with map	.208	.171	.103	53.96	51.95	15.96
Rotate from memory	.209	.201	.065	21.00	19.16	6.72
Move from memory	.136	.135	.057	10.00	10.03	1.13
Chase	.191	.149	.107	9.58	9.52	1.41
Chase with map	.321	.309	.101	15.17	15.75	3.72

Means, medians, and standard deviations for both performance (i.e., the combined spatial error measure; see Eq 8) and time for each of the eight simple tasks.

**Fig 6:**
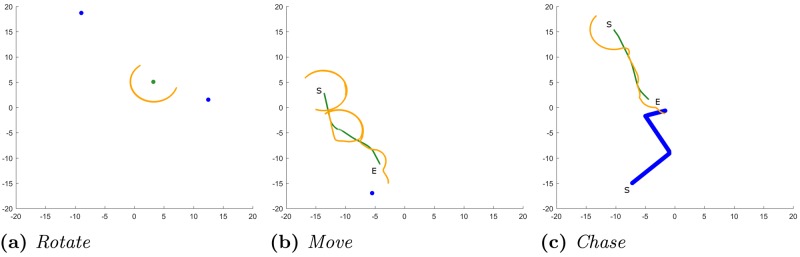
Three exemplary trial results from the simple task phase. The blue circle indicates the target sphere. The green circle/line indicates the position of the participant. The orange line indicates the viewing direction via the location of the catchment area. If a participant or sphere moves, the *S* indicates the starting location and the *E* indicates the end location. (a) Rotation from Memory (RFM): The participant rotates back and forth between two spheres. (b) Move with Map (MWM): The participant rotates in the beginning, moves towards the target, and then rotates again. In the end, the participant is slightly inaccurate with respect to the target’s location. (c) Chase (CHA): The participant rotates in the beginning to find the sphere and then intercepts it.

In terms of both performance and time, participants tended to have more difficulties (i.e., less accurate and slower) with the allocentric and memory tasks than with the egocentric and perceptual tasks. However, these differences must be interpreted with caution because there are exceptions. For example, performance on the rotate task was lower than the rotate with map task, although participants were fastest when completing the rotate task overall. In addition, the rotate, move, and chase tasks were very similar in terms of completion time but exhibit very different spatial error patterns (see Fig 6). For this reason, we will focus the remaining analyses on performance error. Rather than comparing these tasks directly, we will assess them with respect to their abilities to predict navigation performance.

### Relationship between navigation and simple tasks

We performed REFA to attempt to reduce the dimensionality of the data from the eight simple tasks. Permutation tests [64] suggested that the first three factors of the REFA were significant. A simple structure [71, p. 140ff] for a factor analysis reduces dependency between the factors by rotating all of them by the same amount. A rotated factor is considered simply structured if some dimensions are zero (or close to zero in a more relaxed form), and better rotations produce the higher number of zero elements in all factors [72, p. 115ff]. With a geomin rotation [68], we obtained three sets of factors that satisfy the simple structure assumption. All three sets equally represent the underlying factor solution [72], but we focus on Set 1 for two reasons. First, following the goal of Thurstone’s simple structure assumption [71], we can provide a theoretical interpretation for the underlying factors in Set 1. Second, Set 1 was the only set of factors that produced a significant result under robust regression. See Table 2 for the communalities of all three sets, and Table 3 for the results of the robust regressions.

**Table 2:** Communalities and factors of REFA.

Task	Set 1			Set 2			Set 3			C
	F1	F2	F3	F1	F2	F3	F1	F2	F3	
ROT	−.042	−.392	.522	**.705**	.096	.265	−.406	−.081	.484	.424
MOV	.592	−.024	−.322	−.547	.478	.045	**.619**	.431	−.016	.593
RWM	.536	.546	−.252	**−.768**	−.039	.201	**.827**	−.066	−.198	**.746**
MWM	**.715**	.440	.020	−.546	.047	.458	**.841**	−.119	.083	**.709**
RFM	.043	**−.680**	−.374	.012	**.712**	−.406	−.172	**.788**	.013	**.641**
MFM	**.831**	−.032	.325	−.066	.377	**.624**	**.609**	.061	.501	.599
CHA	−.166	−.086	**−.610**	−.430	.203	−.538	.017	.438	−.434	.339
CWM	.356	**−.619**	−.043	.135	**.717**	−.004	.025	**.610**	.316	.516

The REFA produced three sets, each composed of three significant factors (**F1**, **F2**, **F3**) with wide communalities. Loading strengths above a conservative threshold of .6 [73, p. 101] are in bold. The communalities (**C**) for the three sets were identical because each set represents the same underlying unrotated factor under a geomin rotation.

**Table 3:** Performance measures for participants.

Type	Factor	*χ*^2^	*β*	*p*-value
Confirmatory	Egocentric	2.291	0.810	.130
	Allocentric	0.019	0.051	.890
	Distance	0.050	−0.091	.822
	Direction	0.026	−0.087	.872
	Perceived	0.950	0.485	.330
	Remembered	0.113	0.152	.737
	Static	0.055	−0.124	.815
	**Dynamic**	**5.473**	**0.800**	**.019**
Exploratory	Set 1 F1	0.399	−0.110	.527
	Set 1 F2	.968	−0.212	.325
	**Set 1 F3**	**10.550**	**−0.850**	**.001**
	Set 2 F1	0.716	−0.142	.397
	Set 2 F2	0.900	0.183	.343
	Set 2 F3	0.880	−0.442	.348
	Set 3 F1	0.009	−0.009	.923
	Set 3 F2	1.082	0.213	.298

Results of the robust regressions for both confirmatory (i.e., theoretically defined) and exploratory (i.e., derived using REFA) analyses. With a Šidák correction (*p* < .006), only Set 1 F3 (composed of only the chase task) significantly predicts the mean distance error ratios.

We considered loading strengths above a conservative threshold of.6 [73, p. 101]. According to Jung and Lee [64], the factor analysis resulted in a relatively wide range of communalities (from .339 to .746; see Table 2). Total communality (*h*_*t*_ = 4.567) indicated that our factors explain 57.1% of overall variation in participants’ performance. Three of the tasks resulted in high communality (rotate with map, move with map, and rotate from memory) above the high threshold of .6, and four of the other tasks resulted in communalities above the low threshold of .4 (rotate, move, move from memory, and chase with map). The chase task was the only task with a communality below .4 (.339), suggesting a low correlation with each of the other tasks.

**Fig 7:**
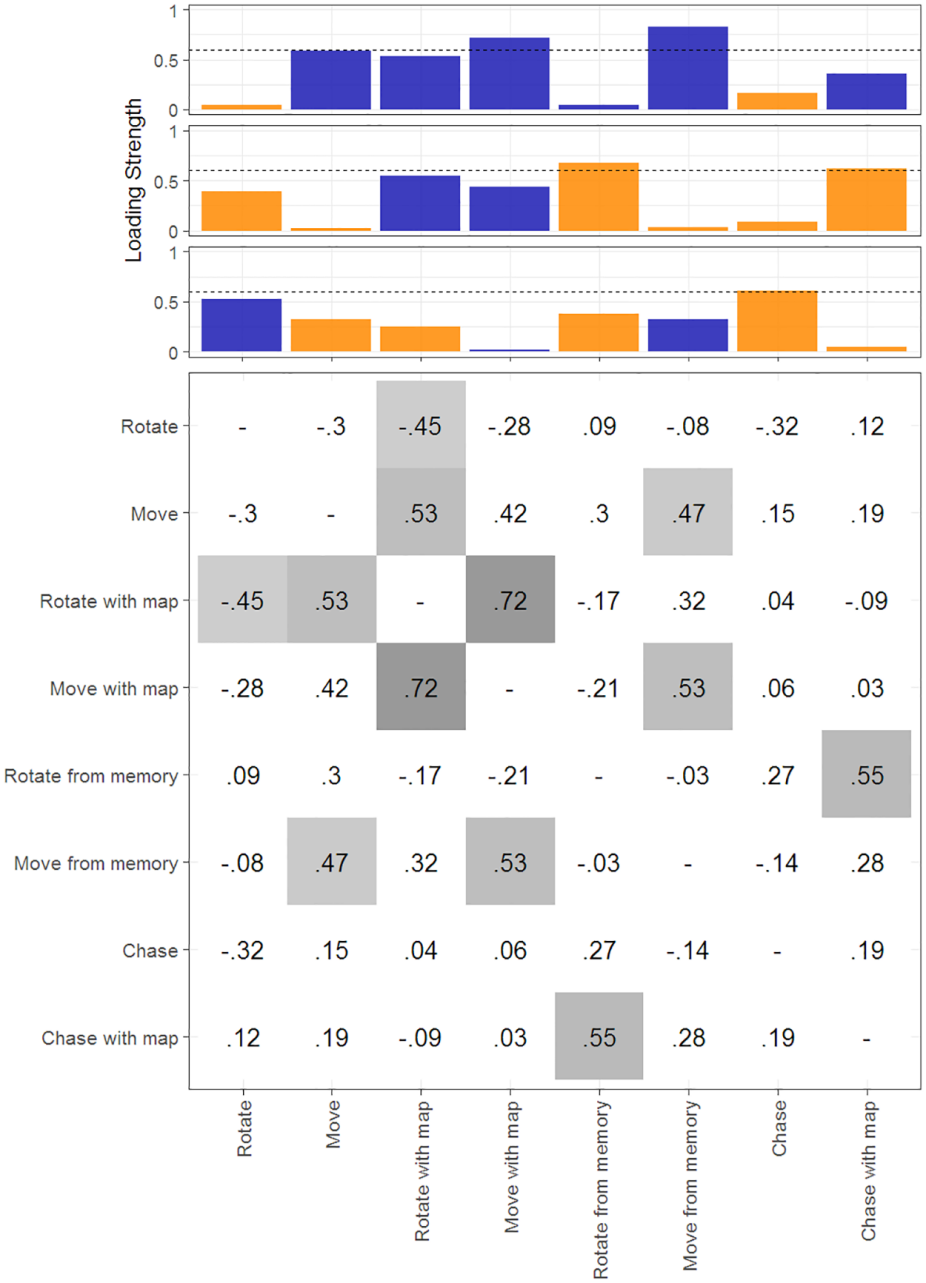
Correlations and REFA factors. This visualisation exhibits the first set of REFA factor loadings at the top and the correlation matrix at the bottom. Each column in the top part corresponds to the same column in the matrix. The loading strengths of each factor are colour-coded according to whether they are positive (blue) or negative (orange). The conservative loading threshold of .6 [73] is shown as a dashed line, and any loading above that threshold is considered meaningful. In the correlation matrix, any significant correlation (*p* < .05, *abs*(*ρ*_20_) = .42) is coloured. The visualisation is based on [74] (see S2 Code).

The REFA results for Set 1 exhibit two notable patterns that are also reflected in the correlation matrix of performances on the eight simple tasks (Fig 7). First, the rotate from memory and chase with map tasks both have high loadings for the second factor for each REFA set. The correlation between rotate from memory and chase with map performances is also significant (*r*_18_ = .549, *p* = .012). Second, the chase task is the only task that did not correlate with any other tasks (all *ps* > .16) and is also the only task with a high loading for factor three of the first REFA set. Move from memory is the only task with a high loading for factor three of the third REFA set, although move from memory was significantly correlated with move (*r*_18_ = .466, *p* = .039) and move with map (*r*_18_ = .517, *p* = .017).

We then performed separate regressions for predefined categories of tasks (e.g., egocentric, perceived) and each of the REFA factors on the mean distance error ratios from the Navigation Phase. White’s test for heteroscedasticity [75] revealed that the residuals resulting from the regression of the third REFA factor from the first set on mean distance error ratios was heteroscedastic (*r*^2^ = .37, *White* = 7.47, *p* = .02). Individual White’s tests on the residuals of all other predefined and REFA factors (for all three sets) were not significant (all *ps* > .2). For consistency, we used robust regressions to test for relationships between each predefined and REFA factor and mean distance error ratios. Robust regressions for the effects of seven of the eight predefined factors on the mean distance error ratios were not significant (see Table 3). However, a robust regression for the effect of mean performance for the dynamic tasks on the mean distance error ratios was significant ($\chi_{1}^{2}=5.47$, *β* = 0.80, *p* = .019, uncorrected for multiple comparisons). Additional robust regressions for the effects of each REFA factor of each set were not significant (see Table 3), except for the third factor of the first set ($\chi_{1}^{2}=10.55$, *β* = .85, *p* = .001). This relationship survives a Šidák correction [76] for alpha inflation (*α* = .016). Consistent with the significant effect of dynamic tasks on mean distance error ratios, this REFA factor represents only the chase task.

## Discussion

In this study, we investigated the relationships between eight spatial tasks and navigation performance in virtual reality (VR). These eight tasks were designed in accordance with four orthogonal dimensions based on previous research (static/dynamic, [37]; perceived/remembered, [14]; egocentric/allocentric, [30]; distance/direction, [56]). This approach was adopted in order to provide evidence for or against particular two-systems theories and to determine whether theories of navigation can be reduced to one predictor or require additional factors. Together with this confirmatory analysis, we also attempted to reduce the dimensionality of the model by conducting a regularised exploratory factor analysis (REFA). Both the confirmatory and exploratory factors were then used to predict the participants’ navigation performance with robust regressions. The confirmatory analysis determined that only the dynamic factor (composed of chase and chase with map tasks) significantly predicted navigation (uncorrected for multiple comparisons). In addition, the exploratory analysis revealed that the chase task by itself was the only significant predictor after a Šidák correction. These results suggest that navigation in VR may be best explained by a dynamic, egocentric task that requires the perception of distances and directions.

Unlike previous studies [77], we explicitly devised an error score that accounts for both accumulated and final errors for all eight simple tasks, see Eq 8. This error score includes accumulated error as a means of revealing the process of solving the task. For example, participants sometimes accumulated large errors in the static direction tasks by rotating in place more than was necessary before responding. Such behaviour would not have been detected by considering only the final error. Because we weighted the various error score components (see Eq 8), no advantage was given to the dynamic tasks. At the same time, cumulative error was necessary for scoring the dynamic tasks given that they required the continuous integration of distance and direction information.

Previous research has largely neglected dynamic spatial tasks and has focused instead on tasks in static environments in which only the user moves [1, 32–34]. This work has been critical for investigations of spatial memory but may overemphasise the role of representation (compared to the role of direct perception) during navigation [12]. Our results suggest that, even in a familiar environment, a dynamic chase task that relied primarily on locomotion was a better predictor of navigation performance than typical measures of spatial memory (e.g., distance and direction estimation). Because participants could not have predicted the direction of the target sphere’s movement, the chase task did not rely on spatial memory. Rather than implying that spatial memory is not important for navigation (as in [12]), participants most likely developed representations that were relatively basic but consistent with each other. At the same time, participants’ performances on the chase task required the coordination of visual input with the manipulation of the HID and may have been more variable. Similar to homing in aviation, the interception of a randomly moving sphere required the observer to orient so that the target was at the centre of the expanding optic flow [36]. Instead of spatial memory, the chase task relied on a combination of perceived distances and directions, which is typical of locomotion in real environments.

The extent to which a chase task may predict navigation through a real environment has yet to be investigated independent of spatial memory. Conceptually, such a chase task could resemble the avoidance of other people in crowded environments during locomotion (e.g., [78, 79]). For example, Moussaid and colleagues [79] have developed a cognitively inspired model of pedestrian dynamics in order to explain crowd phenomena such as spontaneous lane formation. These experiments constitute an important aspect of research in spatial cognition but have not been studied in the context of large-scale navigation. Future studies could relate the avoidance of crowds to navigation behaviours (e.g., route choice) in a large public space (e.g., a shopping mall).

In real environments, locomotion is nearly automatic because walking is typically learned at an early age and continuously reinforced. However, the interaction between the user and a virtual environment is mediated by a human interface device (HID). Indeed, this additional layer of abstraction must be learned before users can efficiently interact with the virtual environment [5, 80]. For example, McKinley, McIntire, and Funke [80] found that expert video game players can control a virtual unmanned aerial system to a similar level as trained pilots and better than people with little to no gaming experience. This pattern of performances suggests that prior experience with an HID (for both pilots and gamers) can facilitate interaction with a virtual environment.

In the context of navigation, individual differences in users’ abilities to mannoeuvre with an HID may confound differences in spatial learning. In other words, inferences regarding the development of spatial representation with navigation experience in VR may sometimes be attributable to participants’ abilities to interact with an HID. The relationship between HID interaction and navigation performance may be especially relevant when the virtual environment is over-learned. In the present study, participants were highly familiar with the virtual environment before the beginning of the testing block of the Navigation Phase. This is indicated by the monotonic decrease in trigger presses across training blocks. Indeed, some participants were able to complete the third training block without calling the guiding arrow.

Future studies should ensure that participants are well-trained with the HID and that their abilities to use the HID is properly assessed. Training may reduce the HID’s impact on navigation performance in VR, while assessment can allow researchers to draw inferences regarding spatial learning. Here, our chase task and cumulative error may be especially useful. This approach may also be used for ambulatory VR setups (e.g., treadmills, [81]; large-tracking spaces, [82]). These setups have the advantage of more realistic control over the observer’s movement by providing proprioceptive feedback [83]. For example, Kearns and colleagues [83] found that optic flow can be sufficient for solving a triangle completion task with a joystick, but proprioceptive feedback during walking reduced variability in participants’ responses. Despite this advantage, most ambulatory VR setups are limited in space or still require the user to adapt their gait (e.g., walking in place, [84]; redirected walking, [85]). As such, training and assessment with an HID may be necessary for any experiment involving navigation in VR.

## Supporting information

S1 File Instructions for participants. Text handed out to the participants before the experiment.
(ODT)

S1 Video Summary of tasks in all phases. In four minutes, we show extract of all the phases the participants completed and show exemplary tasks within the phases.
(MP4)

S1 Data Database export. Export of the participant data, ready for loading into Matlab.
(MAT)

S2 Data CSV data set. Transformed data ready for analysis in statistical software such as R or SPSS.
(CSV)

S1 Code Matlab code. Code used for data processing in Matlab.
(ZIP)

S2 Code R script for correlation/loading visualisation. Short script to visualise the factor loadings and correlation matrix based on the design used by [74] and adapted for our purpose. Detailed instructions on how to create such a visualisation can be found at http://rpubs.com/danmirman/plotting_factor_analysis.
(R) Click here for additional data file.
