# Supplementary figures and images for: Evaluation of a conceptual framework for predicting navigation performance in virtual reality
Source: PLoS One. 2017 Sep 15;12(9):e0184682. doi: 10.1371/journal.pone.0184682 (PMC5600378; doi:10.1371/journal.pone.0184682)

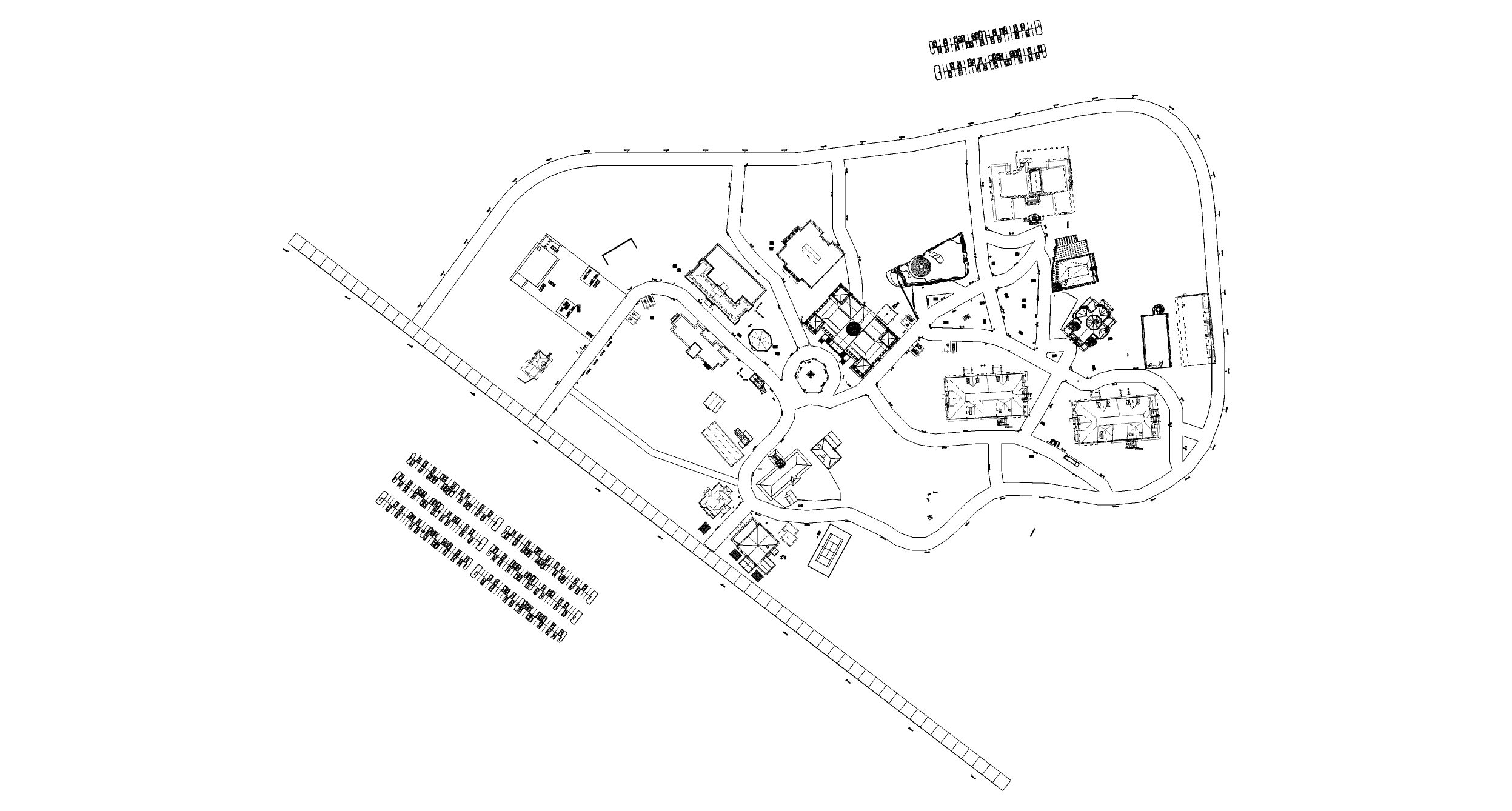

Supplement: S1 Code — Code used for data processing in Matlab. (ZIP) [file pone.0184682.s005.zip › S1_Code_Matlab/ambler_wireframe.png]
