# Supplementary material for: Evaluation of a conceptual framework for predicting navigation performance in virtual reality
Source: PLoS One. 2017 Sep 15;12(9):e0184682. doi: 10.1371/journal.pone.0184682 (PMC5600378; doi:10.1371/journal.pone.0184682)
Supplement: Supplementary file 18 [file pone.0184682.s001.odt]

Today you participate in a two stage experiment. In both stages you will be using a CAVE automated virtual environment. The CAVE consists of 3 screens and will use 3D display technology. The system is designed to be immersive. Some people experience motion sickness with such a system. In case you feel unwell immediately inform the instructor and the experiment will be terminated. You will receive your payment, even if you cannot finish the experiment.

It is crucial for the experiment that you fully understand your tasks and are able to perform them. Therefore please read through the instructions carefully and address any questions you have to the instructor. At the end of the description there are several questions for you to answer. Once you answered all the question the experiment will begin.

First Stage

General Overview:

You are situated on a small square, surrounded by 20 white spheres. The first stage consists of 8 tasks repeated 11 times. The first 8 repetitions are for training.

You will have a minimal interface consisting of text info (called hint) in the top left corner telling you what task you currently have to perform and a map that will be displayed for certain tasks. Further whenever a task starts the task will be displayed shortly in the center of the screen.

You will perform two types of action:

- Look at spheres marked blue

- Walk to spheres marked blue

Whenever you have to walk to a sphere, you will see a yellow circle marking the area before you. You reached the sphere you have to walk to, when the sphere is inside of the yellow circle.

The variations in the tasks will be described here, later on you will get a quick overview of the specific tasks:

For some tasks the sphere is only marked on the map and not in the world. Your task is to identify the sphere on the map in the world and either walk to it or look at it.

For some task the spheres will disappear and you will have to perform the task from memory.

For some tasks the spheres will move around.

Detailed task description:

- You have to look at the sphere which is marked blue. Please do not move, just rotate towards it. When you face the sphere with your head, not the main screen, you should press the trigger on the joystick to finish the task. The hint will be “Look at”. The task is introduced with the following message in the center of the screen: “Look at the marked sphere”.

- In this task the map appears. You see one sphere marked blue on the map and you have to look at it in the world. Please do not move, just rotate towards where you think the sphere is, once you face it,  you should press the trigger on the joystick to finish the task. The hint will be “Look at”. The task is introduced with the following message in the center of the screen: “Look at the sphere marked on the map”.

- You have to walk to the sphere which is marked blue. When the sphere is inside the yellow marker you should press the trigger to finish the task. The hint will be “Walk to”. The task is introduced with the following message in the center of the screen: “Walk to the marked sphere”.

- In this task the map appears. You see one sphere marked blue on the map and you have to walk to it in the world. When the sphere, you think is marked on the map, is inside the yellow marker you should press the trigger to finish the task. The hint will be “Walk to”. The task is introduced with the following message in the center of the screen: “Walk to the sphere marked on the map”.

- This task consists of three parts. First you look two times in a row at a marked sphere, then all spheres disappear and you have to look at where you think the first sphere was.

  - You have to look at the first sphere which is marked blue. Please do not move, just rotate towards it. When you face the sphere with your head, not the main screen, you should press the trigger on the joystick to finish the task. The hint will be “Look at”. The task is introduced with the following message in the center of the screen: “Look at the first marked sphere”.

  - You have to look at the second sphere which is marked blue. Please do not move, just rotate towards it. When you face the sphere with your head, not the main screen, you should press the trigger on the joystick to finish the task. The hint will be “Look at”. The task is introduced with the following message in the center of the screen: “Look at the second marked sphere”.

  - All spheres disappear and you have to rotate from your memory to where you think the first sphere was displayed.  Please do not move. When you face with your head, not the main screen, the place where you think the sphere was you should press the trigger on the joystick to finish the task. The hint will be “Look at”. The task is introduced with the following message in the center of the screen: “Look at where you think the first sphere was”.

- You have to walk to the sphere which is marked blue. After you move more than a meter, all spheres disappear. You may rotate freely before you start moving. When the place, where you think the sphere was, is inside the yellow marker you should press the trigger to finish the task. The hint will be “Walk to”. The task is introduced with the following message in the center of the screen: “Walk to where you think the marked sphere is”.

- In this task the spheres are animated and move around the square freely. You have to walk to the moving sphere which is marked blue. When the sphere is inside the yellow marker you should press the trigger to finish the task. The hint will be “Walk to”. The task is introduced with the following message in the center of the screen: “Walk to the marked sphere”.

- In this task the spheres are animated and move around the square freely, further the map appears. You see one moving sphere marked blue on the map and you have to walk to it in the world. To ease navigation, a red arrow is displayed on the map that indicates your position and orientation. When the moving sphere, you think is marked on the map, is inside the yellow marker you should press the trigger to finish the task. The hint will be “Walk to”. The task is introduced with the following message in the center of the screen: “Walk to the sphere marked on the map”.

Second Stage

General Overview:

You are situated in a small town. The second stage consists of visiting 8 locations throughout the town. Each location will be visited 5 times.

To complete your task your interface consists of four elements on the main screen. In the top left corner you have a hint that states the name of the location you should go to.  In the middle of the main screen a message will be shown whenever you have to go to a new destination. In the top middle part of the screen an energy bar resides, that shows you how often you can call the compass. In the lower middle of the screen the compass is located. The compass is a 3D arrow that points into the direction of your next location to visit. The compass is invisible unless you press the trigger on the joystick. While pressing the trigger on the joystick the energy bar decreases. If the energy bar is nearly empty the compass cannot be triggered. The energy bar slowly refills.

To find a location, you can call the compass by using the trigger on the joystick. You should only use the compass when you are not sure were to go.

You should not walk to close to building (i.e. closer than 2 meters) as objects closer to than the distance from you to the screens might not be properly displayed.

Controls

You get a joystick to navigate the virtual worlds. While the joystick supports many buttons, only a few are in use for this experiment. Please read the instructions carefully to be able to use the controls:

- Moving Forwards: Push the joystick away from you.

- Moving Backwards: Pull the joystick towards you.

- Moving Sidewards: Push the joystick to the left make a step to the left or side or to the right to make a step to the right side.

- Turning around: Twist the joystick to the left to turn leftwards in the virtual world or to the right to turn rightwards in the virtual world.

- You always walk in the direction you look. Looking into another direction will change your walking direction.

- Action: The only action you can take is pressing the trigger on the joystick. Depending on the stage you indicate that you have performed a task (first stage), or you can call the compass to orientate yourself (second stage).

Well-Being

In case you feel sick, please tell the instructor immediately. The screen will be turned black and you may take of 3D glasses and go outside to breathe fresh air. The experiment terminates and you do not have to continue.

Questions

You are currently in the first stage, answer the following questions.

- Your hint states “Look at”, when do you press the trigger?

- You see a map with a red arrow drawn on it, which task do you have to perform?

- In which tasks can the spheres around you disappear?

- What other hint than “Look at” can you get?

- What do you have to do when the spheres are moving??

You are currently in the second stage, answer the following questions

- When is the compass visible?

- How far away from buildings should you remain?

- What happens if the energy bar is empty?
